# Predictors of renal function decline in patients with gastroenteropancreatic neuroendocrine tumors undergoing [177Lu]Lu-DOTA-TATE therapy

**DOI:** 10.1186/s13550-025-01305-8

**Published:** 2025-08-21

**Authors:** Felix L. Herr, Christian Dascalescu, Victoria Fusch, Caroline Smith, Ricarda Ebner, Mathias J. Zacherl, Florian Eckenweber, Konrad Klimek, Christoph J. Auernhammer, Christine Spitzweg, Jens Ricke, Maurice M. Heimer, Clemens C. Cyran, Rudolf A. Werner, Gabriel T. Sheikh

**Affiliations:** 1 Department of Radiology, LMU University Hospital, LMU Munich, Marchioninistr 15 81377, Munich, Germany; 2 Department of Nuclear Medicine, LMU University Hospital, LMU Munich, Munich, Germany; 3 The Russell H. Morgan Department of Radiology and Radiological Sciences, Division of Nuclear Medicine and Molecular Imaging, Johns Hopkins University School of Medicine, Baltimore, Maryland United States; 4 Interdisciplinary Center of Neuroendocrine Tumors of the GastroEnteroPancreatic System (GEPNET-KUM, ENETS certified Center of Excellence), LMU University Hospital, LMU Munich, Munich, Germany; 5 Department of Medicine IV, LMU University Hospital, LMU Munich, Munich, Germany

**Keywords:** GEP-NET, [177Lu]Lu-DOTA-TATE, KDIGO 2024, Renal toxicity, Age

## Abstract

**Background:**

Peptide receptor radionuclide therapy (PRRT) with [177Lu]Lu-DOTA-TATE is an established treatment for advanced gastroenteropancreatic neuroendocrine tumors (GEP-NETs). While overall renal safety is high, the kidneys remain an organ at risk. This study aimed to determine whether clinical parameters can predict the risk of PRRT-associated renal function decline.

**Results:**

This retrospective single-center study included 178 patients with well-differentiated GEP-NETs (Grade 1 or 2) who completed four cycles of [^177^Lu]Lu-DOTA-TATE between 2012 and 2023. Mean baseline eGFR was 81.1 ± 16.3 mL/min/1.73 m² and remained stable at follow-up (81.1 ± 17.8 mL/min/1.73 m², *p* = 0.989). A KDIGO-defined renal function decline (eGFR follow-up to baseline ratio < 0.8) was observed in 15 patients (8.9%). Higher age at baseline was significantly associated with increased risk (OR: 1.07, 95% CI: 1.01–1.14, *p* = 0.023), while baseline eGFR (OR: 1.03, 95% CI: 0.99–1.06, *p* = 0.1) and estimated renal radiation dose (eRRD) (OR: 1.06, 95% CI: 0.89–1.21, *p* = 0.456) were not significant predictors. No significant associations were found for preexisting renal disease, arterial hypertension, diabetes mellitus, or nephrotoxic drugs. ROC analysis yielded an AUC of 0.683 for age, identifying 68.77 years as the optimal threshold for risk stratification of CKD-progression free survival.

**Conclusions:**

While the overall risk of renal function decline following [^177^Lu]Lu-DOTA-TATE therapy of GEP-NET patients is low, age at baseline emerged as a simple yet clinically meaningful predictor of renal function decline in this cohort.

## Background

Peptide receptor radionuclide therapy (PRRT) with [^177^Lu]Lu-DOTA-TATE is a well-established and effective treatment for advanced gastroenteropancreatic neuroendocrine tumors (GEP-NETs). The pivotal NETTER-1 trial demonstrated significantly improved progression-free survival (PFS) in midgut NET patients treated with [^177^Lu]Lu-DOTA-TATE, which lead to consecutive regulatory approval [1, 2]. Subsequent large-cohort studies confirmed durable disease control (median PFS ~ 2–3 years) and favorable overall survival with PRRT​ [2, 3]. PRRT is generally well tolerated; however, its administration entails potential toxicity to non-target organs, among which the kidneys and bone marrow are considered the dose-limiting critical organs​ [4].

Renal toxicity is a particular concern in PRRT because radiolabeled somatostatin analogues are partially reabsorbed by proximal tubular cells, exposing the kidneys to increased activity ​ [5]. Without precautions, this can potentially result in chronic radiation nephropathy, characterized by a progressive decline in glomerular filtration. To mitigate this risk, nephroprotective amino acid infusions (e.g. lysine/arginine) are co-administered to competitively inhibit tubular uptake of the radiotracer​ [6, 7]. In clinical trials and practice, renal function is typically tracked by serial serum creatinine or estimated glomerular filtration rate (eGFR), and progression of chronic impairment is graded according to KDIGO 2024 definition (eGFR ratio follow-up/baseline < 0.8 [8]).

Given the importance of preserving renal function in NET patients, who often achieve prolonged survival, identifying patients at risk for PRRT-induced nephrotoxicity is of high clinical relevance. It is hypothesized that pretherapeutic clinical parameters can predict which patients will develop significant renal dysfunction after PRRT, but there is currently limited data on this topic. In this single-center retrospective study of GEP-NET patients undergoing four cycles of [^177^Lu]Lu-DOTA-TATE, we aimed to determine whether clinical parameters at baseline can predict renal function decline according to KDIGO 2024 definition.

## Methods

### Study design

This retrospective single-center study was approved by the local ethics committee (approval number 19–027). Due to the retrospective nature, informed consent was waived. All consecutive patients with histologically confirmed, well-differentiated GEP-NET (Grade 1 or 2) who underwent four cycles of [^177^Lu]Lu-DOTA-TATE peptide receptor radionuclide therapy (PRRT) at our ENETS-certified Center of Excellence for GEP-NET between 2012 and 2023 were evaluated. A total of 178 patients were included.

The indication for PRRT was established by an interdisciplinary, ENETS-certified GEP-NET tumor board, which convenes weekly and reviews approximately 800 patient cases annually. Board decisions were based on clinical and laboratory markers morphologic tumor progression on contrast-enhanced CT, and SSTR expression as assessed by SSTR-PET/CT.

Inclusion criteria were histologically verified NET G1 or G2, measurable disease on SSTR-PET/CT, and completion of four PRRT cycles.

### Clinical and laboratory parameters

Demographic and clinical parameters included age, baseline renal function, estimated renal radiation dose (eRRD), preexisting renal disease, arterial hypertension, diabetes mellitus, and concurrent use of nephrotoxic drugs. Baseline renal function was assessed using serum creatinine and estimated glomerular filtration rate (eGFR, calculated using the CKD-EPI formula [9]). Follow-up renal function was recorded after completion of all four PRRT cycles. Based on previously reported absorbed renal doses for [^177^Lu]Lu-DOTA-TATE (0.65 Gy/GBq) [10], we calculated the eRRD from the activity administered.

CKD-progression free survival was defined as the number of days between baseline imaging and the occurrence of renal function decline, operationalized according to the KDIGO 2024 guidelines as a ≥ 20% reduction in eGFR from baseline to follow-up [8].

### Statistical analysis

All statistical analyses were performed using R version 4.3.0 (R Foundation for Statistical Computing, Vienna, Austria). Continuous variables are reported as mean ± standard deviation (SD) or median with interquartile range (IQR), as appropriate. Normality was assessed using the Shapiro–Wilk test. Depending on distribution, comparisons between groups were performed using independent t-tests or Mann–Whitney U-tests. Categorical variables were compared using Fisher’s exact test. Correlations between continuous variables were analyzed using Pearson’s or Spearman’s correlation, depending on data distribution. The derived eRRD in Gy, calculated from dosimetry-based renal radiation exposure, was assessed as a continuous predictor for renal toxicity. To identify predictors of renal impairment at follow-up, univariate logistic regression models were applied. Since only one variable reached statistical significance in univariate logistic regression analysis, no multivariate model was performed Odds ratios (ORs) with 95% confidence intervals (CIs) and corresponding *p*-values were reported. To assess the predictive accuracy of baseline variables, receiver operating characteristic (ROC) analysis was conducted for age. The area under the curve (AUC) was calculated, and optimal threshold values were identified using the Youden index. Kaplan–Meier analysis was used to estimate CKD-progression free survival, stratified by the ROC-derived thresholds for age. Survival curves were compared using the log-rank test. Additionally, representative individual patient cases were visualized to exemplify differences in renal outcomes based on these thresholds.

## Results

### Patient characteristics

The patient characteristics are presented in Table 1. The primary tumor was located in the small bowel (*n* = 98/178, 55%), pancreas (*n* = 37/178, 21%), cancer of unknown primary (*n* = 21/178, 12%), ileocecal junction (*n* = 11/178, 6%), large bowel/rectum (*n* = 9/178, 5%), and other locations (*n* = 2/178, 1%). According to baseline SSTR-PET/CT, the metastatic sites identified were the liver (*n* = 144/178, 81%), lymph nodes (*n* = 108/178, 61%), mesenterial/peritoneal (*n* = 108/178, 61%), bone (*n* = 105/178, 59%), and other locations (*n* = 24/178, 13%). Among all patients, *n* = 168/178 (94%) patients received the planned maximum of four PRRT cycles.

**Table 1:** Baseline characteristics of the study population

	*n* = 178
Male to female at baseline	108 / 70
Mean age at baseline mean (SD)	63.5 years (10.6)
Baseline to follow-up after four cycles	10.9 months (11.8)
Administered cumulative activity (GBq) mean (SD)	27.6 (4.8)
Estimated renal radiation dose (Gy) mean (SD)	17.9 (3.1)
**Clinical risk factors** n (%)	
Preexisting renal disease^a^	54 (30)
Arterial hypertension	69 (39)
Diabetes mellitus	14 (8)
Nephrotoxic drugs^b^	76 (43)
Baseline eGFR mean (SD)	81.1 ml/min/1.73 m^2^ (16.3)
Follow-up eGFR mean (SD)	81.1 ml/min/1.73 m^2^ (17.8)
**Primary tumor location**, n (%)	
Small bowel	98 (55%)
Pancreas	37 (21%)
Cancer of Unknow Primary	21 (12%)
Ileocecal junction	11 (6%)
Large bowel / Rectum	9 (5%)
Other	2 (1%)
**Tumor grade**, n (%)	
NET G1 (Ki-67 ≤ 2%)	54 (30%)
NET G2 (2 < Ki-67 ≤ 20%)	119 (67%)
Unknown	5 (3%)
**Metastases**, n (%)	
Liver	144 (81%)
Lymph nodes	108 (61%)
Mesenterium / Peritoneum	108 (61%)
Bone	105 (59%)
Other	24 (13%)
**Therapy Approach before** PRRT *, n (%)	
Surgical resection of primary tumor	105 (59%)
Long-acting somatostatin analogue	110 (62%)
Local ablative liver therapies	49 (28%)
Chemotherapy or everolimus	30 (17%)
Bone-targeted radiotherapy	6 (3%)

*some patients received more than one treatment modality before PRRT

SD = standard deviation; PRRT = peptide receptor radionuclide therapy; BMI = body mass index; NET = neuroendocrine tumor; a Prior CKD, urinary dysfunction, nephrectomy, atrophic kidney or kidney cysts;b Analgetics, antihypertensive medication, lipid lowering medication, furosemide intake

### Longitudinal assessment of renal function during PRRT

Among patients with GEP-NET undergoing PRRT, the median baseline serum creatinine was 0.90 mg/dL and remained stable at follow-up after four cycles of PRRT, with a median value of 0.90 mg/dL (*p* = 0.648). Similarly, the estimated glomerular filtration rate (eGFR) showed no significant change over time, with a median baseline value of 81.99 mL/min/1.73 m² compared to 81.74 mL/min/1.73 m² at follow-up (*p* = 0.989). Patients received a cumulative activity of 27.6 ± 4.8 GBq leading to an eRRD of 17.9 ± 3.1 Gy at follow-up. No significant correlation was found between eRRD and the relative change in GFR at follow-up (*r* = 0.07, *p* = 0.38). At follow-up, a CKD progression according to the KDIGO 2024 definition (eGFR ratio follow-up/baseline < 0.8 [8]) was observed in 15 patients (8.9%), while 153 patients (91.1%) did not meet this threshold for a significant progression of renal function decline.

### Prediction of CKD development under PRRT

To provide an overview of baseline differences between patients with and without relevant renal function decline according to KDIGO 2024 definition (eGFR follow-up to baseline ratio < 0.8), we conducted unadjusted group comparisons. Patients with a GFR ratio < 0.8 were significantly older at baseline compared to those with stable renal function (mean 69.6 ± 9.9 vs. 62.6 ± 10.7 years, *p* = 0.02). In contrast, baseline eGFR was lower in the affected group (74.4 ± 17.9 vs. 82.1 ± 16 ml/min/1.73 m²), but this difference did not reach statistical significance (*p* = 0.127). Similarly, the eRRD was not significantly associated with GFR ratio decline (17.3 ± 3.7 vs. 18.2 ± 2.8 Gy, *p* = 0.446). None of the binary clinical risk factors, including preexisting renal disease (*p* = 0.383), arterial hypertension (*p* = 0.586), diabetes mellitus (*p* = 0.327), or the use of nephrotoxic drugs (*p* = 0.423), showed significant associations with renal impairment. Supporting this observation, Pearson correlation analysis revealed a statistically significant inverse correlation between age at baseline and follow-up eGFR (*r* = − 0.41, *p* < 0.001), indicating that increasing age was associated with a greater likelihood of relevant renal function decline according to KDIGO 2024 criteria.

**Fig. 1:**
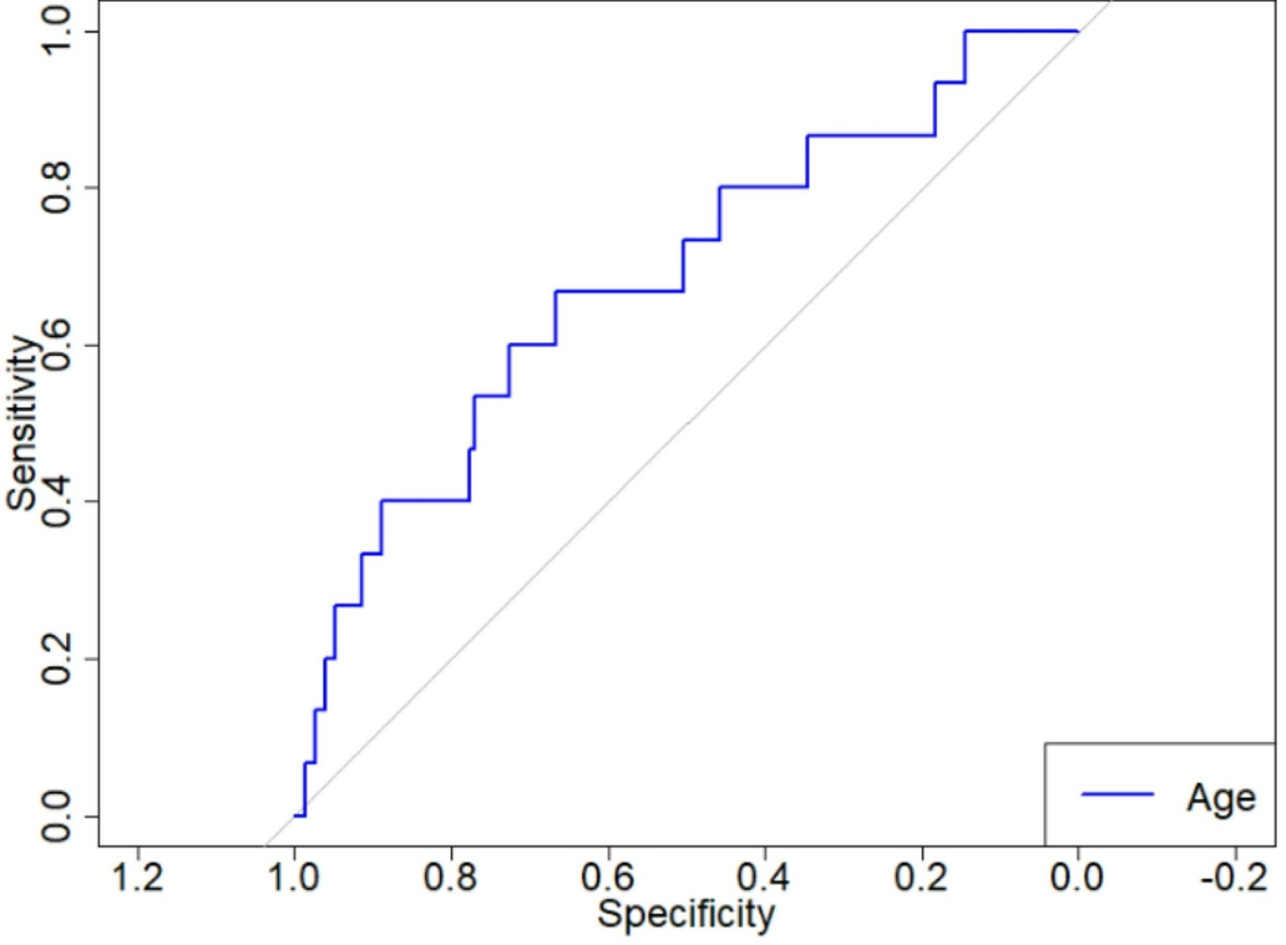
Receiver Operating Characteristic (ROC) curve evaluates the predictive ability of age at baseline for the development of GFR impairment according to KDIGO 2024 criteria. The AUC was 0.683, indicating moderate discriminatory performance. The optimal cut-off value determined by the Youden index was 68.77 years

**Fig. 2:**
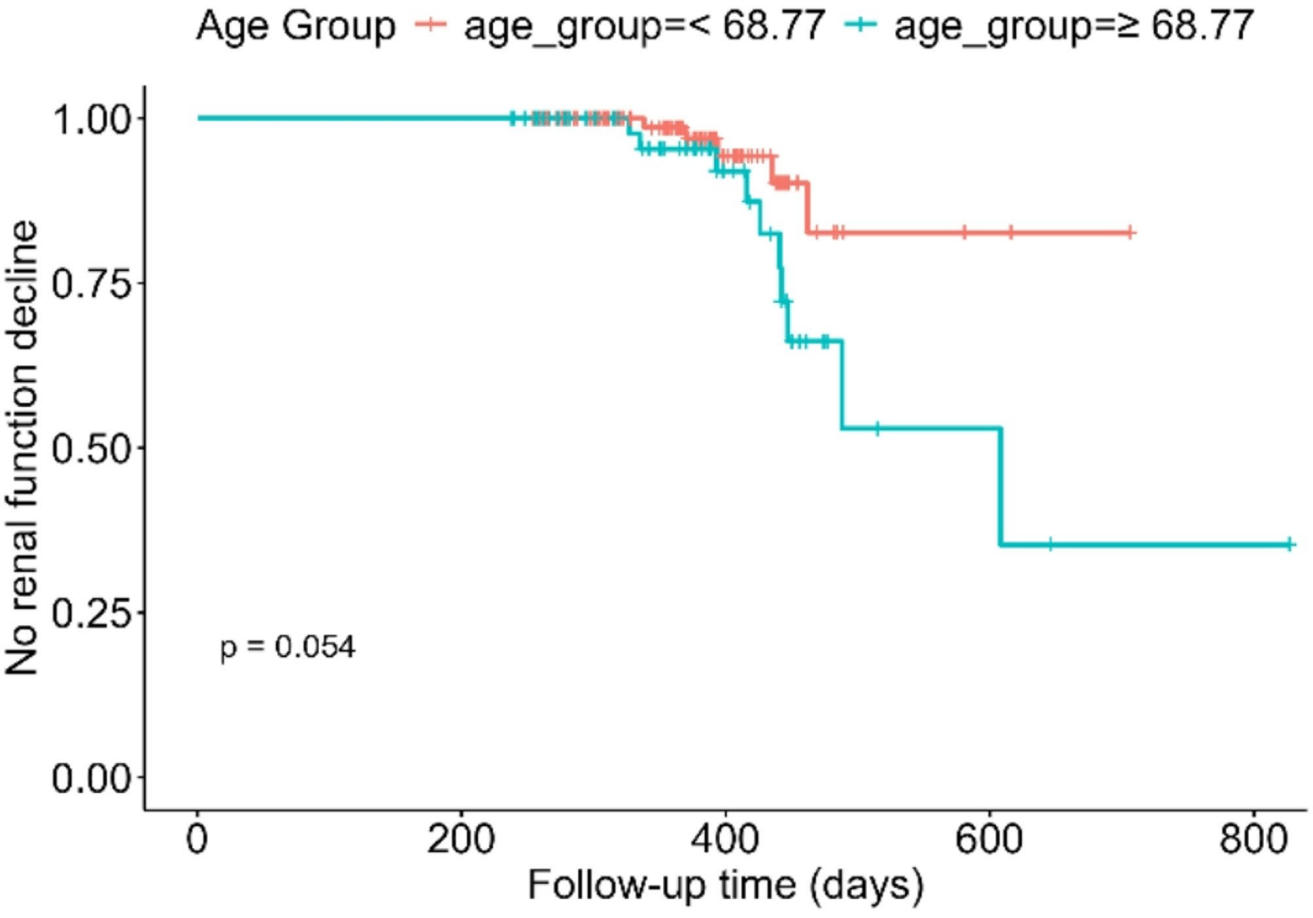
Kaplan–Meier analysis evaluating predictors of KDIGO-defined renal function decline at follow-up. Using the ROC-derived optimal age threshold of 68.77 years, Kaplan–Meier curves were generated to assess GFR impairment-free survival. Patients aged ≥ 68.77 years exhibited a shorter time interval free from KDIGO-defined renal function decline compared to younger individuals. This difference did not reach statistical significance (log-rank *p* = 0.054). Median GFR impairment-free interval was 608 days in the older group, while the median was not reached in the younger group. Survival was defined as the number of days between baseline and follow-up assessment

ROC analysis was conducted to evaluate the predictive ability of age at baseline for the development of KDIGO-defined renal function decline, operationalized as a decrease in follow-up eGFR of less than 80% of baseline (Fig. 1). The analysis yielded a moderate discriminatory performance with an AUC of 0.683. Based on the Youden index, the optimal age cut-off was determined to be 68.77 years. Using this threshold, a Kaplan–Meier analysis was performed to estimate CKD-progression free survival stratified by age group. Patients aged ≥ 68.77 years showed a shorter interval without renal function decline compared to patients aged < 68.77 for whom the median renal function decline free survival was not reached during the observation period. Although this trend suggests a potential association, the difference did not reach statistical significance (log-rank *p* = 0.054). The time interval between baseline imaging and the occurrence of the endpoint — a KDIGO-defined renal function decline — was calculated for each patient and used to define CKD-progression free survival in days (Fig. 2).

To assess individual risk factors associated with a KDIGO-defined renal function decline univariate logistic regression analyses were performed for several baseline clinical parameters (Table 2). Among the tested predictors, higher age at baseline was significantly associated with increased odds of developing KDIGO-defined renal function decline at follow-up (OR: 1.07, 95% CI: 1.01–1.14, *p* = 0.023). None of the other variables demonstrated a statistically significant association with the endpoint. Higher baseline eGFR (OR: 1.03, 95% CI: 0.99–1.06, *p* = 0.1) and higher eRRD (OR: 1.06, 95% CI: 0.89–1.21, *p* = 0.456) were not statistically significant predictors of renal impairment. Similarly, no significant associations were observed for binary clinical factors such as preexisting renal disease (*p* = 0.398), arterial hypertension (*p* = 0.513), diabetes mellitus (*p* = 0.419), or the use of nephrotoxic drugs (*p* = 0.387). Given that only age at baseline showed statistical significance in univariate analysis, no multivariate analysis was performed. These results suggest that older age is a predictor for KDIGO 2024-defined renal function decline in this patient cohort.

**Table 2:** Predictors of CKD in logistic regression analysis

Variable	Univariate Analysis OR (95% CI)	*P*
Age at baseline	1.07 (1.01–1.14)	0.023
Baseline eGFR	1.03 (0.99–1.06)	< 0.1
eRRD	1.06 (0.89–1.21)	0.456
Preexisting renal disease	1.60 (0.51–4.68)	0.398
Arterial hypertension	1.43 (0.48–4.16)	0.513
Diabetes mellitus	1.94 (0.28–8.16)	0.419
Nephrotoxic drugs	1.60 (0.55–4.76)	0.387

eGFR = estimated glomerular filtration rate; eRRD = estimated renal radiation dose; OR = odds ratio; CI = confidence interval; CKD = chronic kidney disease

## Discussion

In our retrospective analysis applying the KDIGO 2024 criteria for binary evaluation of renal function decline after four cycles of [^177^Lu]Lu-DOTA-TATE therapy in patients with GEP-NET, baseline age emerged as the only significant predictor. Older patients were more likely to experience a ≥ 20% decrease in eGFR from baseline, suggesting age-related vulnerability to renal deterioration under PRRT. In contrast, neither baseline eGFR nor eRRD showed a statistically significant association with KDIGO-defined renal function decline, although patients with lower baseline eGFR and slightly lower eRRD values tended to be more frequently affected. Similarly, conventional clinical risk markers such as preexisting renal disease, arterial hypertension, diabetes mellitus, and the use of nephrotoxic drugs were not associated with increased risk. These findings indicate that while PRRT is generally well tolerated from a nephrological standpoint, older age may serve as a practical and clinically relevant risk factor for identifying patients at higher risk for renal decline.

Across the cohort, renal function remained stable during the course of therapy. No cases of severe nephrotoxicity (eGFR < 30mL/min/1.73 m²) or abrupt GFR deterioration were observed during PRRT. Longitudinal eGFR measurements showed minimal changes in most patients. This aligns with earlier reports suggesting that PRRT-associated nephrotoxicity, when it occurs, tends to follow a gradual course rather than causing immediate kidney injury [11]. After four cycles of PRRT, only a small proportion of patients met the criteria for KDIGO-defined renal function decline, and these changes were generally modest. Our results corroborate prior observations of mild, slow eGFR declines—typically in the range of 2–4 mL/min per year—after [177Lu]Lu-DOTA-TATE treatment [11, 12]. Alsadik et al., for instance, reported an average annual decline of 2.5% in patients with preexisting CKD, with nearly half of patients experiencing no significant deterioration over time.

Overall, our findings add to the literature affirming the renal safety of [^177^Lu]Lu-DOTA-TATE. Prior large-scale studies, including those by Brabander et al. [13] and Bergsma et al. [11], reported no instances of severe long-term nephrotoxicity and only rare mild increases in serum creatinine. The cumulative renal dose administered in standard PRRT regimens (approximately 23–29 GBq of [^177^Lu]Lu-DOTA-TATE, resulting in 20–25 Gy absorbed dose to the kidneys) appears to be well tolerated in most patients without preexisting advanced renal disease [6, 14]. Our findings, indicating a mean renal absorbed dose of approximately 19 Gy, lie below commonly cited nephrotoxicity thresholds and may thus explain the limited association between eRRD and renal function decline observed in our analysis. Moreover, the absence of statistical significance could reflect the limited sample size, potentially constraining the ability to detect subtle dose-dependent effects. Importantly, we extend prior findings by identifying baseline age as a clear stratifier of risk even within this generally low-risk population. While earlier work, such as that by Bergsma et al., did not pinpoint predictors of toxicity—likely due to the low number of events in their cohort—we used KDIGO-defined renal function decline as a clinically relevant endpoint and were able to demonstrate meaningful associations [11].

While [^177^Lu]Lu-DOTA-TATE is generally well tolerated by the kidneys, it is clear that those who do experience a clinically relevant decline in kidney function under PRRT are often patients with already marginal renal reserve to start with. The identification of ROC-derived thresholds for age may aid in pre-treatment risk assessment, as older patients seem to have lower tolerance toward renal injury. Age showed value for stratification: patients aged ≥ 68.77 years were more likely to experience renal decline, which can be used to group patients into higher and lower risk categories, thereby informing treatment decisions and follow-up strategies.

### Limitations

This study is limited by its retrospective single-center design, which may introduce selection bias and limit generalizability. The relatively low number of patients with significant renal impairment at follow-up restricts the statistical power for subgroup analyses. Additionally, long-term renal outcomes beyond the follow-up window of this study remain unknown.

## Conclusions

In this large cohort of GEP-NET patients undergoing [^177^Lu]Lu-DOTA-TATE therapy, although renal function remained stable in the majority of patients, older age could be identified as a predictor for subsequent renal impairment. Our findings support the use of age as a simple and clinically relevant tool to stratify the risk of CKD progression after PRRT.

## Data Availability

The datasets generated during and/or analysed during the current study are available from the corresponding author on reasonable request.

## Abbreviations

AUC: Area under the curve
CI: Confidence interval
CKD: Chronic kidney disease
CKD-EPI: Chronic kidney disease epidemiology collaboration
DOTA-TATE: DOTA-Tyr³-octreotate
eGFR: Estimated glomerular filtration rate
ENETS: European neuroendocrine tumor society
GEP-NET: Gastroenteropancreatic neuroendocrine tumor
KDIGO: Kidney disease: improving global outcomes
NETTER: Neuroendocrine tumor therapy (NETTER-1 trial)
OR: Odds ratio
PET: Positron emission tomography
PRRT: Peptide receptor radionuclide therapy
ROC: Receiver operating characteristic
SD: Standard deviation
SSTR: Somatostatin receptor
